# Automated Exposure Notification for COVID-19

**Published:** 2022-12-20

**Authors:** Leo Samuels, Novak Boskov, Andreas Francisco Oliveira, Edwin Sun, David Starobinski, Ari Trachtenberg, Manan Monga, Mayank Varia, Ran Canetti, Anand Devaiah, Gerald V Denis

**Affiliations:** 1 Biological Sciences, University of Maryland, College Park, 20742, MD; 2 Department of Electrical and Computer Engineering, Boston University, 8 St. Mary’s St, Boston, 02215, MA; 3 Department of Computer Science, Boston University, 111 Cummington Mall, Boston, 02215, MA; 4 School of Medicine, Boston University, 72E Concord St, Boston, 02118, MA

## Abstract

In the current COVID-19 pandemic, various Automated Exposure Notification (AEN) systems have been proposed to help quickly identify potential contacts of infected individuals. All these systems try to leverage the current understanding of the following factors: transmission risk, technology to address risk modeling, system policies and privacy considerations. While AEN holds promise for mitigating the spread of COVID-19, using short-range communication channels (Bluetooth) in smartphones to detect close individual contacts may be inaccurate for modeling and informing transmission risk. This work finds that the current close contact definitions may be inadequate to reduce viral spread using AEN technology. Consequently, relying on distance measurements from Bluetooth Low-Energy may not be optimal for determining risks of exposure and protecting privacy. This paper’s literature analysis suggests that AEN may perform better by using broadly accessible technologies to sense the respiratory activity, mask status, or environment of participants. Moreover, the paper remains cognizant that smartphone sensors can leak private information and thus recommends additional objectives for maintaining user privacy without compromising utility for population health. This literature review and analysis will simultaneously interest (i) health professionals who desire a fundamental understanding of the design and utility of AEN systems and (ii) technologists interested in understanding their epidemiological basis in the light of recent research. Ultimately, the two disparate communities need to understand each other to assess the value of AEN systems in mitigating viral spread, whether for the COVID-19 pandemic or for future ones.

## INTRODUCTION

As COVID-19 cases surged across the world, health authorities implemented contact tracing systems to understand how the virus spread between humans and how to mitigate its spread. Traditional methods use infected individuals’ information to identify at-risk contacts and inform local health departments that implement exposure and quarantining protocols with health monitoring. To augment this process, electronic methods of contact tracing were developed, but researchers quickly realized their potential for leaking privacy-sensitive metadata (Minami and Borisov, 2010; Scheck, 2010; Tiwari et al., 2019; Valentino-Devries et al., 2018). This risk of private information being leaked also discourages participation in electronic contact tracing (Ries, 2020). As a result, several groups proposed privacy-aware Automated Exposure Notification (AEN) systems based on anonymous Bluetooth Low-Energy (BLE) short-range communications (Canetti et al., 2020; Chan et al., 2020; Rivest et al., 2020; Trieu et al., 2020; Troncoso et al., 2020). Google and Apple then collaborated to create the Google/Apple Exposure Notification system (GAEN), which was publicly released in mid-May 2020 (Google, 2020).

AEN systems typically utilize the reception of wireless Bluetooth signals to assess one’s physical proximity within the range of presumed viral transmission (e.g., up to 2 m apart for at least 15 minutes (CDC, 2020)). In a common implementation, AEN-enabled phones broadcast random numbers (called tokens) and receive similar tokens from nearby broadcasters. Users diagnosed with COVID-19 direct their phones to anonymously upload recently transmitted tokens to a public token registry, which other users regularly check to determine whether they may or may not have been infected. One of the key assumptions underlying modern AEN systems is that the proximity and duration of contacts are primary determinants of viral transmission.

This study investigates the basis for this assumption, starting with a reverse citation search from contemporary scientific articles related to viral transmission and AEN. Of approximately 120 articles found, articles with either low relevance (based on primary subject matter) or low impact (based on citation count and source reputation) were excluded. Out of necessity, due to space constraints, our summaries in this study are brief, but the reader is invited to delve further into the cited works to better understand the claims presented. One overarching observation from the reverse citation search is that the biological and epidemiological foundations of current definitions for “close contacts” show that a broader approach may be needed to improve automated exposure systems. For example, many recent studies have shown that individuals can be infected by respiratory droplets containing viral material traveling great distances while suspended in air, meaning that proximity may be a poor metric on its own for assessing transmission risk (Bazant and Bush, 2021). As a result, the significant efforts undertaken in modern AENs to extract reliable distance information from Bluetooth data may have been misguided (Leith and Farrell, 2020b). This article concludes with recommendations for improving future AEN systems from both technological and epidemiological bases.

AEN is a promising and novel approach for effective and privacy-protecting mitigation of viral spread. To be successful, however, the technologists who develop the AEN system and the scientists who research viral transmission may need to understand the fundamental limitations of their disparate knowledge bases more deeply.

### Current Contact Definitions

The current definitions of a close contact vary throughout the world. The United States Center for Disease Control (CDC) defines a close contact (as of August 11^*th*^, 2022) as:

“Someone who was less than 6 feet away from an infected person (laboratory-confirmed or a clinical diagnosis) for a cumulative total of 15 minutes or more over a 24-hour period”

(CDC, 2020).

On the other hand, the World Health Organization (WHO) defines a close contact as:

“Anyone who had direct contact or was within 1 metre for at least 15 minutes with a person infected with the virus that causes COVID-19, even if the person with the confirmed infection did not have symptoms”

(WHO, 2020).

The European Centre for Disease Prevention and Control (ECDC) defines a contact most like the CDC as:

“[anyone] having had face-to-face contact with a COVID-19 case within 2 meters and > 15 minutes,”

(ECDC, 2020).

However, the ECDC also includes other criteria to define a contact, such as having direct physical contact with an infected person.

All these close contact definitions appear to be based on early observational studies completed in the 1930s and 40s. For example, a study by Wells et al. in 1934 observed that large, 0–1 mm diameter, emitted respiratory droplets landed, on average, within 3–6 feet of their source. A different study in 1941 found that 0–1mm diameter droplets traveled on average 2–3 feet from their source (Turner et al., 1941). Both papers indicated that smaller droplets expelled from the mouth and nose evaporated into “droplet nuclei,” which are dry, possibly pathogen-carrying respiratory particles that have the ability to travel 6 feet by air currents.

The CDC seems to have added the cumulative 15 minutes over 24 hours exposure rule after an outbreak investigation at a prison in Vermont (Pringle et al., 2020). Before this outbreak, a correctional officer spent one minute each with six incarcerated individuals while separated 6 feet apart and wearing full protective gear. However, the correctional officer developed COVID-19, and the CDC confirmed the guard had not interacted with anyone infected in the 14 days before he became sick. This CDC guideline suggests that viral transmission risk may not necessarily depend on exposure at one instance of 15 minutes but could be additive through shorter exposures over a 24-hour period.

## MODERN ANALYSIS OF RESPIRATORY DROPLET SPREAD

Although early studies provided support for the current distancing guidelines, they assumed a simple dichotomy of droplet size: small or large. In reality, there is a wide spectrum of droplet sizes up to 1mm that travel different distances based on their size and external conditions (Bowen, 2010).

### Distance

Using high-speed imaging technologies, researchers visualized expelled droplets and found that larger droplets fell within 1–2 meter (m) diameters, whereas smaller ≤ 50*μ*m diameter droplets evaporated much quicker yet stayed suspended in the air within 6–8m (Bourouiba et al., 2014). Using a model simulation, one group found that larger droplets ≥ 50*μ*m, from sneezing, traveled more than 6m (Xie et al., 2007). Another analysis found that ≤ 30*μ*m saliva droplets are weakly affected by gravity and can travel more than 2m (Zhu et al., 2006). As many have demonstrated, droplets can travel well beyond 2m.

### Infectivity

A central question about which droplets can carry infectious virus particles remains. One experiment collected aerosol samples from six COVID-19 patients and found viable (capable of replicating) SARS-CoV-2 RNA in aerosols ≤ 5*μ*m (Santarpia et al., 2020). However, this study did not explore how long these aerosols can remain infectious. Through investigating the duration of viability, one study found viable SARS-CoV-2 RNA in aerosols which had remained in the air for 3 hours (Van Doremalen et al., 2020). Researchers further wanted to examine the viability of SARS-CoV-2 compared to other viruses. Comparing the stability of SARS-CoV-2 to SARS-CoV and MERS-CoV, scientists discovered that SARS-CoV-2 remained viable for a longer time period, with some lasting up to 16 hours (Fears et al., 2020). Through investigating viral aerosol viability with different respiratory actions, one study compared the number of viable influenza particles from coughing and breathing in 53 influenza-infected subjects and concluded that 53% produced viable influenza particles from coughing and 42% from breathing (Lindsley et al., 2016). These works suggest that viral aerosols may be capable of remaining infectious after being expelled and for relatively long time periods.

### Aerosols

While large droplets appear to primarily fall within 2m, smaller aerosols that can remain suspended for longer may provide a primary route for viral transmission. While attempting to explain the prevalence of aerosols, one experiment discovered that violent exhalations are mainly composed of a turbulent, moist gas cloud that may allow droplets to avoid evaporation longer than isolated droplets (Bourouiba, 2020). The research on the sizes of respiratory droplets that travel more than 2m differs in its findings. While one study found that most expelled droplets had diameters ≤ 2*μ*m (Johnson and Morawska, 2009), another study discovered a slightly larger expelled droplet majority of 6*μ*m (Chao et al., 2009). A different paper determined that 1–10*μ*m droplets were much more prevalent than larger 100–1000*μ*m droplets in the cough of healthy human subjects. Moreover, these smaller droplets were only found when subjects spoke (Somsen et al., 2020). In contrast, while these studies focused on the respiratory emissions of healthy people, a different study compared exhalations of healthy versus sick individuals. Hersen et al. analyzed the breath of 78 volunteers, 43 of whom had respiratory illnesses and 35 of whom were healthy (2008). The symptomatic volunteers emitted larger concentrations of respiratory particles than the healthy volunteers, especially around the 0.5*μ*m aerosol size. Furthermore, another study found that particle emission rate is positively correlated with speech frequency and volume (Asadi et al., 2019). While the literature debates the majority size of emitted droplets, the research agrees that humans exhale many small aerosols.

The literature thus suggests that the majority of emitted droplets are small aerosols that are capable of carrying viable viruses further than 2m and remain suspended in the air for longer periods of time. As a result, AEN systems utilizing close-contact guidelines solely focusing on 2m distancing and time may be missing other potentially significant pathways of viral transmission.

## REAL-WORLD LIMITATIONS OF CURRENT GUIDELINES

Real-world examples of aerosol transmission, mask use effectiveness and the ways by which environmental factors impact droplet spread may also inform our understanding of defining a close contact for COVID-19.

### Aerosol Transmission

As presented above, the current epidemiological literature suggests that humans generate small aerosols that carry infectious viruses and travel further than 2m. There are many instances where small aerosols may have played a dominant role in infection ( ; Guy, 2021) (Li et al., 2020; Park et al., 2020; Shen et al., 2020). One of these instances occurred in March 2020 when a 61-member choir practiced for 2.5 hours, and one person was symptomatic of COVID-19 (Hamner, 2020). The choir participants were socially distanced, but 87% of the group developed COVID-19, possibly because the symptomatic person emitted infectious aerosols that spread throughout the room. Although there is no direct evidence to measure whether or not aerosols caused the choir or other outbreaks, Hamner’s article suggests credible mechanisms of how small particles could provide a route of viral transmission.

There has also been work done simulating the risk due to potential virus-carrying aerosol particles. Bazant and Bush recently showed how to calculate the cumulative exposure time (CET) before a person reaches the risk of contracting the virus (2021). For a well-mixed room and a constant number of viral aerosols, this CET is proportional to room volume, the airflow rate, and the filtration efficacy of the masks worn. Furthermore, it is inversely related to the infectiousness of air particles and the relative viral transmissibility. Hence, larger rooms with better ventilation and higher mask efficacy lead to lower aerosol contraction risk. The choir incident investigation suggested that aerosols may play a significant role in infection, and the research on simulations of ambient conditions may also guide an improved automated determination of infection risk.

### Masks

Understanding mask filtration efficacy and their utility in reducing viral transmission are needed in order to assess the value of incorporating them within an AEN system.

#### a) Masks and Particle Spread

One of the most cited studies on masks involved 246 individuals with respiratory illnesses who were randomly assigned to either wear a mask or not for 30 minutes. For unmasked participants, 26–30% of emitted droplets (> 5*μ*m) and 35–56% of aerosols (≤ 5*μ*m) contained viral coronavirus, influenza and rhinovirus RNA. For masked participants, no coronavirus droplets or aerosols were found, although 4–38% of influenza and rhinovirus RNA was found in both droplets and aerosols (Leung et al., 2020). Similarly examining exhaled virus, another study collected and measured influenza RNA from 37 volunteers while masked or unmasked. They concluded that surgical masks (i) almost blocked coarse > 5 *μ*m particles, (ii) reduced fine particle emission 2.8-fold and coarse particles 25-fold and (iii) overall decreased particle shedding by 3.4-fold compared to no masks (Milton et al., 2013). While these two studies focused on droplets larger or smaller than 5 *μ*m, Papineni and Rosenthal in 1997 specifically found that the most ubiquitous particles exhaled from a P100 mask were 1*μ*m, 2*μ*m and 8*μ*m. Lastly, exploring the efficacy of masks in general, one analysis found that while many masks block the forward momentum of cough droplets, air escapes from the sides, even when wearing a tightly fitted N95 mask (Tang et al., 2009). Overall, these studies suggest that using masks may decrease the risk of particle spread, which may be relevant for AEN systems depending on the size and number of particles needed to transmit infection.

#### b) Inhalation

While many mask studies focus on the filtration efficiency for exhaling particles, several works investigate mask filtration performance for inhaling particles. In one study, N95 masks showed a filtration efficiency of 89.6%, surgical masks 33.3%, bandanas 11.3% and dust masks 6.1% (Bowen, 2010). Another study found that surgical masks may reduce influenza virus inhalation by 1.1x to 55x, with an average of 6x, depending on the mask design (Booth et al., 2013). As a result, face masks may filter exhalations more effectively than inhalations, with some types of masks being more capable than others. These studies suggest that mask-wearing detection might be a useful consideration for AEN.

#### c) Real-World Evidence of Mask Use

In community settings, mask effectiveness appears to be a function of various factors. One study compared the efficacies of different mask types in public health settings, randomly assigning medical or cloth masks to 1,607 healthcare workers in Hanoi, Vietnam. Infection rates and lab-tested particle penetration were significantly higher for cloth masks than for medical masks (Macintyre et al., 2015). Further comparing different masks, researchers found that N95 masks significantly lowered respiratory infection rates among 1,441 Beijing healthcare workers, but medical masks did not do the same (Macintyre et al., 2011). On the other hand, one analysis found no significant differences in efficacy between N95 and cloth masks among 2,371 healthcare workers during peak respiratory illness season (Radonovich et al., 2019). Another paper discovered comparable results through a meta-analysis of 1 randomized trial and 12 full tests (Bartoszko et al., 2020). These conclusions display mixed results for mask effectiveness in reducing viral infection, with some finding certain masks more advantageous for protection, while others finding no difference in safety between mask types.

#### d) Mask Efficacy

Challenges in understanding the variables around community masking and transmission are long-standing and persistent (Kellogg, 1919). Bundgaard et al. (2020) randomly assigned 4,862 people to either wear surgical masks or not in a Danish community with moderate infection rates, some amount of social distancing and infrequent mask usage. The study was powered to observe a 50% infection reduction, but they did not find a statistically significant reduction of COVID cases for those who were masked. By analyzing the effectiveness of physical interventions to reduce infection, other researchers also did not find a reduction in viral transmission (Jefferson et al., 2020). Similarly, after evaluating 35 COVID-19 reports, a study found that face masks did not significantly reduce infection risk (Coclite et al., 2020). Contrarily, one analysis found that when masks were first mandated on April 6th, 2020, COVID-19 cases declined by about 75% after 20 days in comparison to a control group with no masks (Mitze et al., 2020). Another research group tracked COVID-19 infection rates of 9,850 healthcare workers from March 1^*st*^-April 30^*th*^, 2020 and found that COVID-19 cases increased from 0 to 21% with non-mandated mask-wearing but decreased thereafter to 11.46% with masks required (X. Wang et al., 2020). In addition, the CDC associated mask mandates showcased a small but statistically significant decrease in daily growth rates of COVID-19 cases and deaths within 20 days of rule implementation (Guy, 2021). These findings collectively demonstrate the importance of further research into the role of masks as part of a successful mitigation strategy and informing risk models for AEN systems.

### Environment

Small indoor environments with poor ventilation may result in higher COVID-19 risk. A research group found that out of 320 outbreaks in China, 318 occurred indoors (Qian et al., 2020). Similarly, a different group analyzed 25,000 COVID-19 cases and found that only 6% of cases were totally or partially outdoors (Weed and Foad, 2020). Investigating the role of ventilation with various viruses, one research group reviewed and analyzed ten COVID-19 studies and discovered a strong association between building ventilation and airborne infection of SARS, tuberculosis, measles and influenza (Li et al., 2007). Additionally, a study found that the number of droplets halved in 30 seconds in a room with a door and window open, while the droplet number halved in 5 minutes in a closed room (Somsen et al., 2020). These data demonstrate that ventilation and setting can inform risk modeling for AEN systems.

Overall, there is evidence that the current close contact definitions are too narrow to capture SARS-CoV-2 transmission modalities fully. Therefore, by focusing on large droplet transmissions, scientists may be neglecting potentially significant elements of transmission such as small aerosols, mask filtration and environmental conditions. Considering these factors may increase the efficacy of current close contact rules in reducing viral communication.

## TECHNICAL APPROACHES

Next, this study will focus on the ability of AEN systems to prevent COVID-19 outbreaks by reviewing the effectiveness of these systems and their privacy concerns. Motivated by the previous sections describing the challenges of using distance as a measure of risk, this paper reviews two measurements that could aid AEN systems that utilize a more comprehensive definition for a contact.

### Effectiveness

Though it is still unclear whether AEN is an effective approach for reducing virus transmission, there is some early analysis suggestive of efficacy. Researchers evaluated the NHS COVID-19 app launched in England and Wales in September 2020 and found that 16.5 million individuals use the app regularly, which is 49% of the population with compatible phones. The paper’s authors inferred that the app may have helped prevent more than 224,000 infections from October to December 2020 (Wymant et al., 2021). In an unreviewed pre-print, one study examined the effect of the SwissCovid COVID-19 contact-tracing app in Zurich. They found that the app sent quarantine instructions to an equivalent of 5% of exposed contacts that were told to quarantine by manual contact tracing and 17% of those people tested positive (Menges et al., 2021). More data is needed to understand the impact of AEN, but there is some suggestion of its efficacy in identifying viral spread.

### Privacy

Public trust is important for widespread adoption and, hence, the usefulness of AEN applications. To ensure trust, applications must require users’ consent during installation, provide an opt-out option from data collection and be transparent in what information it shares. Additionally, no identifiable information regarding a person should be collected or divulged to any institution until that information is obfuscated and de-identified.

Different applications worldwide have met these guidelines with varying levels of success. For example, China’s application must be used by all citizens, and it also requests considerable personal information (Klar and Lanzerath, 2020). User movement is then restricted based on color-coded access dependent on the application’s evaluation of threat. On the other hand, Iceland’s application, IRakning C-19, has taken clear steps toward user privacy, requiring consent, an opt-out option and automatic deactivation and data deletion after the pandemic. Roughly 38% of eligible citizens utilize this app, and this is a relatively high proportion of the population compared to other countries (Hamilton, 2020).

A broader study conducted by UK researchers found that most applications provide clear information on contact tracing procedures and addresses concerns of users’ privacy, but some do not request user permission for data sharing or disclose which may allow third-parties to access their data (Sun et al., 2021). Moreover, over half utilize at least one deprecated cryptographic algorithm, casting doubts on their ability to guarantee minimal levels of security. Based on the current data, user consent and personal information protection may encourage wider adoption of AEN.

### Distance Measurement

Most modern mobile phones are equipped with Bluetooth Low Energy (BLE), an energy-efficient short-range radio. Bluetooth periodically sends out beacon messages across three different radio channels, allowing other BLE-equipped devices to capture beacon transmissions for evaluation by the operating system. Although one may infer the distance between two phones from the strength of these BLE signals, there are several scenarios that could mask the relationship between true distance and received signal strength. For example, the transmission power of the broadcasting device may vary across devices, as illustrated by some sample measurements collected (Table 1). Two smartphones were set up in an isolated environment, and the Bluetooth of both phones was turned on. As the phones advertise their BLE signals (tokens), the measured Received Signal Strength Indicator (RSSI) values were read on the receiving end, and the minimum and maximum RSSI values were reported over a short time interval. The measurements support that a considerable variation of RSSI is observed, even in controlled settings, and that different devices measure different RSSI at the same distance. Other factors impacting RSSI include occlusive, absorptive, or reflective surfaces, such as furniture or human bodies (since they can affect signal propagation) (Leith and Farrell, 2020b). The angle and polarization of the phone signal can also impact RSSI (Hatke et al., 2020).

To test BLE accuracy, a research group measured the signal strengths of mobile phone BLE for people in three settings: walking outside in a city, sitting at a table and sitting on a train. In the first situation, two individuals walking behind each other with a 1m gap produced similar signal strength to that of two people side by side with a 2m gap. In the second scenario, signal strength between four people sitting around a table decreased significantly when phones were in their owner’s pockets and not on a table. Finally, in a train, the signal strength decreased as people spread apart except when moving from 3.5m to 4m. This decrease was likely due to surface reflections (Leith and Farrell, 2020b).

A similar experiment looked at participants using the Google/Apple Exposure Notification contact tracing app on a European commuter train while placed less than 2m apart for 15 minutes. Signal strength attenuation stayed roughly constant at around 52dB from 1–2.5m apart, then sharply increased at 3m and then decreased to 60dB at 4m. The researchers then applied Swiss, German and Italian detection rules to the app data. They found that the Swiss and German parameters triggered no contact notifications while Italian rules generated a false positive and false negative rate of 50% each. Furthermore, when only evaluating the impact of a human body, a handbag, or walls, they found that signal strength significantly fluctuated when the signal path was altered (Leith and Farrell, 2020b).

Due to the BLE signal strength variability and several factors impacting it, one study demonstrated that ultrasonic sound measurements could supplement BLE implementations and significantly improve the accuracy of the distance estimates. The speed of sound is relatively constant, and two devices can jointly calculate the distance between them with the help of time-tagged (ultrasonic and inaudible) acoustic pulses. Unlike BLE, ultrasound typically does not penetrate walls and other large obstacles, possibly matching virus movement restrictions for better contact estimation. However, receiving ultrasound signals typically requires access to a smartphone’s microphone, which may also capture sensitive information (e.g., speech and background noise) in the process. The privacy risk for this technology must therefore be offset by the potential benefit of establishing a more accurate contact distance (Meklenburg et al., 2020).

Though BLE is ubiquitous in modern smartphones, there are reliability issues with using the technology to consistently measure distance. Future AEN systems may wish to supplement BLE with other technologies, such as ultrasonic sound, if distance computation is a defining feature.

### Indoor – Outdoor

The potential significance of indoor versus outdoor (IO) detection makes it a valuable consideration for AEN applications. Most IO automated detection approaches involve Machine Learning (ML) models on a set of smartphone sensor measurements. For example, learning-based IO detection on Global System for Mobile Communications (GSM) data is 97% accurate on data comparable to the one used to train the model (W. Wang et al., 2016). Unfortunately, the collected data calibrates the model according to the environmental conditions. Hence, in order to produce efficient IO detection across many environments, users would need GSM data that fits all possible environments. To address this issue, a group of researchers developed a semi-supervised online system wherein a user collects initial training data. Two specified ML algorithms are then trained on the data and update themselves on unlabeled data through an online co-training method. This method achieved 91% accuracy in environments in which it had not been trained (Radu et al., 2014). In short, if users are willing to collect (or download) training data, then AEN systems can reasonably rely on IO prediction. Furthermore, if these models are trained on the user’s device and data (which stays solely within the phone), and no communication is made with the AEN app, then IO detection can be privacy-preserving.

### Mask Detection

Although the precise effectiveness of masks is debated, masks have been shown to filter droplets that may be involved in transmission. Thus, it is possible that mask detection may help assess a user’s risk of infection and transmission. To test the effectiveness of mask detection, heuristic experiments were conducted on mask detection by embedding an open-source Bluetooth-enabled Ruuvi device inside a mask. The Ruuvi can detect whether a mask is being worn based on changes in humidity and temperature (in this case, rising to 90% and 30°C, respectively when the mask is worn). Accelerometer data can also help identify a user’s activity, whether walking or coughing more frequently. Others have performed similar experiments on a “smart-mask” with humidity and temperature sensors, including particulate matter (PM) sensors (which detect higher aerosol concentrations, particularly 0.3–0.5*μ*m particles) (Masna et al., 2020). Given its Bluetooth capability, the smart mask can be integrated into an Android application with a machine learning module to categorize the infection risk. Finally, there have been efforts to utilize ubiquitous image recognition techniques in detecting individuals that do or do not wear masks in public spaces. Technologists developed a highly accurate neural network-based mask detector called RetinaFaceMask that utilizes multiple feature maps and feature pyramid networks for object detection (Jiang et al., 2020). Although this method is shown to be highly accurate and efficient, it may raise privacy concerns as it requires image or video input that may involve unconsenting individuals.

### Rollout

Ensuring the public understands the technology at their disposal is crucial to effective adoption. A properly conducted and open educational effort is essential to maximizing the potential of AEN systems. Using trusted voices, such as health care providers and community leaders, to educate the public can also help drive understanding and adoption. Public technology comprehension is especially important because once users reject AEN (and, say, remove the corresponding app), there are questions about whether they will ever install it (or its relations) again. At the very least, a coordinated rollout campaign will help people understand the technology and the choices available, culminating in whether they decide to adopt or decline the use of AEN systems. These systems’ public health, privacy and utility implications are important to communicate in a rollout campaign. Additionally, system activation and deactivation on phones should be taught in these rollout campaigns. Having technical support available to those who have questions is important and can help with any technical problems that may arise after deployment.

## DISCUSSION

AEN systems have shown promise for mitigating viral spread. As previously discussed, in England and Wales, some estimates suggested that AEN may have aided in preventing over 224,000 infections in early 2020 (Wymant et al., 2021). Moreover, AEN sent quarantine instructions to a small percentage of exposed individuals whose potential sickness was confirmed by manual tracing (Menges et al., 2021). The AEN marriage of technology and epidemiology has been designed to utilize short-range radio-frequency communications, common to modern smartphones, as a proxy for tracing the contacts of infectious individuals. Crucially, they can do so while preserving the privacy of the users. However, to be effective, their use must be based on sound epidemiological models, most notably the definition of what may be a possible contact exposure. Additional policies should be built on the current understanding of viral transmission to provide a foundation for further refinement and implementations, thereby making AEN more viable as a public health tool for transmission mitigation.

The current scientific literature on respiratory droplet dissemination, mask efficiency and environmental setting can shed new light on additional parameters for defining a COVID-19 close-contact. The following speculative guidelines may be useful to complement the current system of contact-tracing apps:

1. Guidelines for proper ventilation could be useful in reducing infection risk, as ventilation in indoor environments appears to have a significant effect on droplet and aerosol distribution.
2. Monitoring respiratory activity may be advantageous in determining occupants’ infection risk because different respiratory activities, such as sneezing or coughing, generate varying amounts of differently sized droplets.
3. Proximity to an infected individual (within 6 feet for more than 15 minutes) may not be an effective indicator of viral transmission, as viral aerosols and droplets may spread quite far and even remain suspended in the air. Moreover, though masks may block large exhaled respiratory droplets, they do not appear to effectively intercept smaller aerosols that may be implicated in transmission.
4. The evidence on community mask effectiveness is mixed: some studies found that mask-wearing reduced the number of COVID-19 cases while others found no significant effect. In terms of personal efficacy, masks may efficiently filter exhalations but not inhalations. As such, diagnosis of infection risk may need to consider the mask status of all participants.
5. Inequitable access to technology may amplify the disparities in health care and put vulnerable people at greater risk. The next versions of this technology should be accessible to the broadest possible population, regardless of socioeconomic status or location. This can involve different strategies, such as utilizing widely available and cost-effective hardware platforms and state or federal-level assistance for those needing access to this technology.

In this study, the biological underpinnings of the close-contact definition, which is at the heart of almost all modern exposure notification applications, were investigated. Review of the literature points to two notable conclusions: (i) the current distance-time definitions of close contact may well be insufficient to properly mitigate disease spread through AEN systems and (ii) the focus on accurate distance measurements from Bluetooth-Low Energy communication may be consequently misguided. As a result, several additional technologies were outlined that could be employed to provide privacy-cognizant exposure notification mechanisms that are more effective in mitigating both the current COVID-19 pandemic and future pandemics.

## Figures and Tables

**Table 1.** The Received Signal Strength Indicators (RSSI) between an iPhone 11 and a OnePlus 7 phone. RSSI is an estimated measure of power in dBm (decibel-milliwatts) that one device receives from another. A higher value (closer to 0) indicates a stronger connection. The parentheses in the second and third columns report the minimum and maximum RSSI that the two devices collected from each other at the distances shown in the first column. For the iPhone 11, the RSSI received from the OnePlus 7 varied between −45 and −51 at a distance of 1 foot, was constant at −59 at a distance of 3 feet, and was unable to transmit at a distance of 6 feet. For the OnePlus 7, the RSSI received from the iPhone 11 at 1 foot ranged between −44 to −46, increased to a range of −36 to −46 at a distance of 3 feet and decreased to a range of −54 to −58 at a distance of 6 feet. Thus, different devices report a range of differing RSSI even when measured at the same distance.

	Device Used	
Distance (ft)	iPhone 11	OnePlus 7
1	(−45, −51)	(−44, −46)
3	−59	(−36,−46)
6	N/A	(−54,−58)
